# Influence of Fused Deposition Modeling Process Parameters on Constitutive Model of Hyperelastic Thermoplastic Polyurethane

**DOI:** 10.3390/polym17010026

**Published:** 2024-12-26

**Authors:** Lucas Gallup, Mohamed Trabia, Brendan O’Toole, Youssef Fahmy

**Affiliations:** Department of Mechanical Engineering, University of Nevada, Las Vegas, NV 89154, USA; gallup@unlv.nevada.edu (L.G.); brendan.otoole@unlv.edu (B.O.); fahmy@unlv.nevada.edu (Y.F.)

**Keywords:** fused deposition modeling (FDM), 3D printing, hyperelastic, thermoplastic polyurethane, constitutive models

## Abstract

Thermoplastic polyurethanes (TPUs) are suited for fused deposition modeling (FDM) of parts that require high levels of flexibility and strength. Predicting the deformation of TPU parts produced using FDM may be difficult, especially under large deformations, as their constitutive models depend on the printing process parameters. The lack of understanding led to the absence of constitutive models for TPU parts produced using FDM. This work aims to identify accurate hyperelastic constitutive models. Six groups of uniaxial tensile specimens were produced using FDM. These groups were made with variations in two process parameters, which were infill geometry and extrusion nozzle temperature. Infill geometries either corresponded to a zero-deposition angle (wall-only) or an infill deposition of ±45° raster angle (infill-only). It was determined that a third-order Mooney–Rivlin constitutive model can accurately describe these six groups. A finite element analysis (FEA) of the experiments using the proposed constitutive models resulted in limited errors for all groups. The proposed approach was verified through a combination of experiments and FEA of FDM TPU components undergoing large deformation.

## 1. Introduction

In FDM, a polymer filament is fed into a nozzle at a certain feed rate and melted to create a layer according to the movement of the nozzle. The process is repeated in each layer to create a part. It is well known that FDM process parameters affect the behavior of the printed parts. These process parameters include the feed rate, infill density, infill pattern, build orientation, layer height, print speed, infill geometry, wall thickness, and extrusion nozzle temperature. Additional parameters such as print bed temperature, print chamber temperature, and the location of the part on the print bed affect the quality of printed components. However, not all FDM printers have controlled print bed temperatures or print chamber temperatures. Multiple researchers have addressed this topic [1,2,3,4,5,6,7,8,9,10,11,12,13,14,15,16,17,18,19,20,21,22,23,24,25,26,27,28,29].

A brief overview of relevant research in this area is presented in this section. Infill density is the ratio of printed material to the total volume within a part, indicating the relative amount of solid material and voids. Multiple researchers have shown that it has a clear impact on the mechanical characteristics of 3D printed parts [3,5,12,16,17,20,21,22]. These studies indicate that increasing the infill density led to an increase in Young’s modulus of elasticity, yield, and ultimate strengths. The influence of part orientation on the build plate has been extensively investigated [4,6,7,13,23,24,26]. The results indicate that identical specimens that were printed flat or on-edge showed a significantly higher ultimate stress than the upright specimens [6,7,13,26]. Few researchers have considered the influence of wall thickness on the behavior of FDM parts. For example, the authors of [8] used axial and flexion testing to show that increasing the thickness of the wall region and the number of wall lines resulted in an increase in the percent elongation at failure, tensile strength, Young’s modulus, and toughness of NinjaFlex^®^ specimens [3,8,9,12,16,22].

Two important FDM process parameters are the infill geometry and extrusion nozzle temperature on the mechanical behavior. The following is an overview of some of the most relevant work in these two areas: The influence of material deposition orientation on the compressive strength of acrylonitrile butadiene styrene (ABS) was studied [5]. The results showed that infill geometry did not cause a significant change in compressive Young’s modulus, except when loading occurred parallel to the infill geometry. Similarly, mechanical properties were determined for polylactic acid (PLA) specimens that were printed with three infill geometries, grid, tri-hexagonal, and concentric [12]. The concentric specimens had consistently higher Young’s modulus, yield strength, and ultimate strength, while the tri-hexagonal and grid specimens performed similarly. The elastic modulus and tensile strength of honeycomb and gyroid infill geometry in two print orientations were compared [18]. Honeycomb-style infill geometry was found to result in higher Young’s modulus and tensile strength for both orientations, though the magnitudes of results were of the same order [10]. The relationship between the printer extrusion nozzle temperature and the mechanical characteristics of various materials have been investigated [3,14,27,28,29]. The effect of two independent FDM parameters, layer height and nozzle temperature, on the mechanical behavior of TPU parts was explored using a combination of tensile, flexural, and impact tests [30]. Similarly, the effects of layer thickness, printing speed, and extruder temperature on FDM TPU were studied [31]. Overall, mechanical properties such as Young’s modulus and yield tensile strengths increased with the temperature, plateauing as the temperature reached peak values [27,28].

While these studies have investigated several key FDM process parameters, the combined effect of infill geometry and extrusion nozzle temperature on mechanical properties is not well understood [10]. These two parameters are among the easiest to adjust when setting a 3D printer to create a component. The literature review indicates that no studies consider the effects of these parameters on TPUs, which are a class of polymers typically used for applications requiring large deformation, flexibility, and toughness. While other polymers such as PLA and ABS may have stronger mechanical properties, TPU is significantly more flexible and durable [1].

This study aims to address this issue using NinjaFlex^®^, which is a widely available TPU. These two process parameters, infill geometry and extrusion nozzle temperature, can be easily controlled in most FDM machines, making understanding their influences important when varying the material characteristics is desired. Tensile specimens were created using two geometries, infill-only and wall-only. Infill-only deposition uses a ±45° raster angle in consecutive layers. Wall-only describes the case when the material is deposited along the same direction at each layer. These two deposition methods are commonly available in standard slicer software, and many parts are created with infill-only in the middle and wall-only surrounding it; they are significantly distinct to enable the understanding of their effect on mechanical properties. Three extrusion nozzle temperatures were used. Based on the experimental results, a constitutive model was identified, as well as the effect of the tested process parameters on this model. Results were validated using FEA.

## 2. Materials and Methods

### 2.1. Material and Preparation

**Figure 1:**
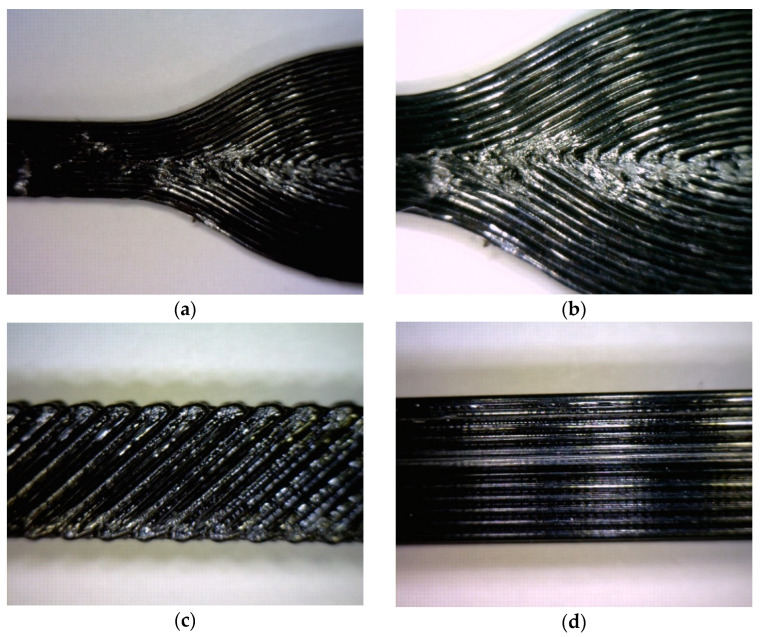
Tensile specimens; (**a**) wall-only dog bone specimen; (**b**) zoomed-in view showing discontinuities in specimen (**a**); (**c**) infill-only specimen; (**d**) wall-only specimen.

NinjaFlex^®^ tensile specimens were prepared using model slicing software Cura LulzBot Edition software Version 4.13.10 (Fargo Additive Manufacturing Equipment 3D, LLC, Fargo, ND, USA) [32]. They were then printed on a Lulzbot Taz Mini 2 [33] with a 0.5 mm Aerostruder^®^ tool head (Fargo Additive Manufacturing Equipment 3D, LLC, Fargo, ND, USA) [31]. Dog bone specimens were initially developed according to ASTM D638-14 [34]. However, this geometry resulted in several discontinuities in the transition between the two cross-sectional areas, as shown in Figure 1a,b. Therefore, the flared ends were removed, leading to the use of long and rectangular specimens. Figure 1c,d shows the infill-only and wall-only specimens. Printing parameters are listed in Table 1, while the test matrix is presented in Table 2. Ten tensile specimens of each group were printed, resulting in a total of 60 experimental specimens. Batches of five infill-only and five wall-only well-spaced specimens were printed on the build plate together.

### 2.2. Experimental Setup

**Figure 2:**
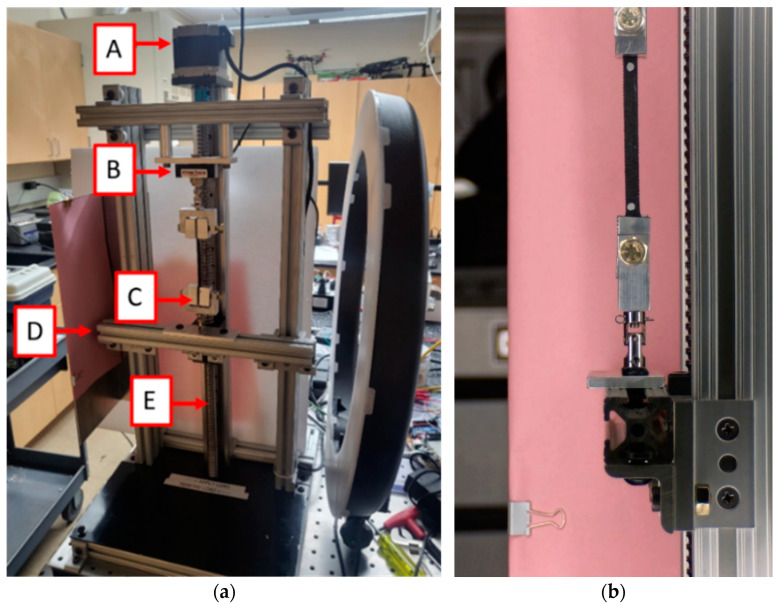
Tensile testing experiment; (**a**) low-force uniaxial tensile testing machine, (A) actuating DC motor, (B) load cell, (C) specimen in grips, (D) linear rail system, (E) ball screw; (**b**) specimen while being loaded.

The specimens were tested on a custom-built low-force uniaxial tensile testing machine, Figure 2a. The testing machine, which was attached to an optical table, used a stepper motor in conjunction with a ball screw and linear rail system to apply a specified displacement rate. A 25 lb (111 N) load cell [35] measured the tensile force at a rate of 30 samples per second using a National Instrument Data Acquisition System (DAQ) with LabView^®^ (Austin, TX, USA). All parts in the tensile testing machine and the fixture support joints are metallic and therefore significantly stiffer than the tested material. Any slack in the system was accounted for and removed during the data processing stage. The elongations of the specimens were recorded with a Back-Bone Modified GoPro Hero 10^®^ camera (San Mateo, CA, USA) and a Nikon AF-S Nikkor^®^ 18–140 mm Lens (Tokyo, Japan) at a rate of 30 frames per second. The camera was located 1.5 m to the right of the tested specimens. The videos were recorded at a 5.3 k resolution or 15.8 MP for each frame. After testing multiple background colors, it was found out that a pink backdrop behind the specimens best reduced the optical noise, Figure 2b.

The dimensions of the tensile specimen can be found in Table 3. These dimensions were based on several factors as follows:

1. ASTM638-14 states that the minimum width of a tensile specimen is 6 mm.
2. A specimen thickness of 3 mm was based on combining the maximum force of the load cell, the ultimate strength of NinjaFlex^®^ [36], and the minimum width specified in ASTM638-14o.
3. Based on the camera setup, it was aimed to choose a free length that can achieve an engineering strain of 2, which is equivalent to a 1.1 true strain, without deteriorating the quality of captured images. This strain range is within what has been reported by other researchers [8,15,37].

The experiments were conducted at a displacement rate of 0.2 mm/s. This rate was slow enough to ensure quasi-static conditions while still allowing for the collection of significant experimental data. The following summarizes the testing procedure: after the tensile specimens were placed in the grips, circular paint markers were added to both ends of the specimen near the grips, Figure 2b. Next, grips with the installed specimen were placed in the tensile machine.

### 2.3. Data Processing

**Figure 3:**
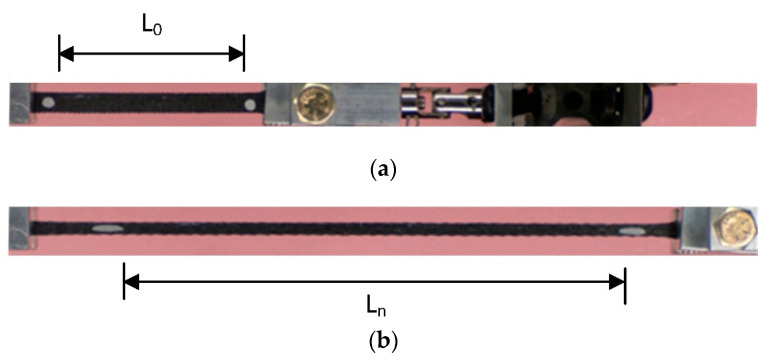
Typical phases of the tensile testing experiment; (**a**) specimen initial image; (**b**) specimen final image.

Frames were extracted from the videos of all experiments. These frames were cropped based on the initial and final images in each experiment, Figure 3. The approximate image resolution is 24 pixels/mm. A displacement rate of 0.2 mm/s can be converted to approximately 4.8 pixels/s. The camera collected data at 30 fps, which resulted in excessive quantities of data. To reduce the processed data without affecting the quality of the experiment, one in every 50 frames was used for data processing. Close to 400 frames were typically used in each experiment. These images were converted to grayscale and then binarized. Two markers were identified in each image, and the coordinates of the innermost points were recorded and used to monitor the extension of the specimen. Figure 3a,b shows images of a specimen at the initial and final images. The distance between the markers in these two images is denoted as $L_{0}$ and $L_{n}$, respectively. Similarly, the minimum width of the specimen was identified in each frame. Load cell data were synchronized with the video recording. Finally, true stress and true strain values were calculated at each frame for each experiment.

## 3. Results

### 3.1. Experimental Results

The measured dimensions of the printed specimens of each group can be found in Table 4. A void ratio was included by comparing the average to nominal mass for each specimen group. Nominal mass was calculated based on the nominal volume and the manufacturer’s reported density of 1.19 g/cc [36], assuming no voids.

**Figure 4:**
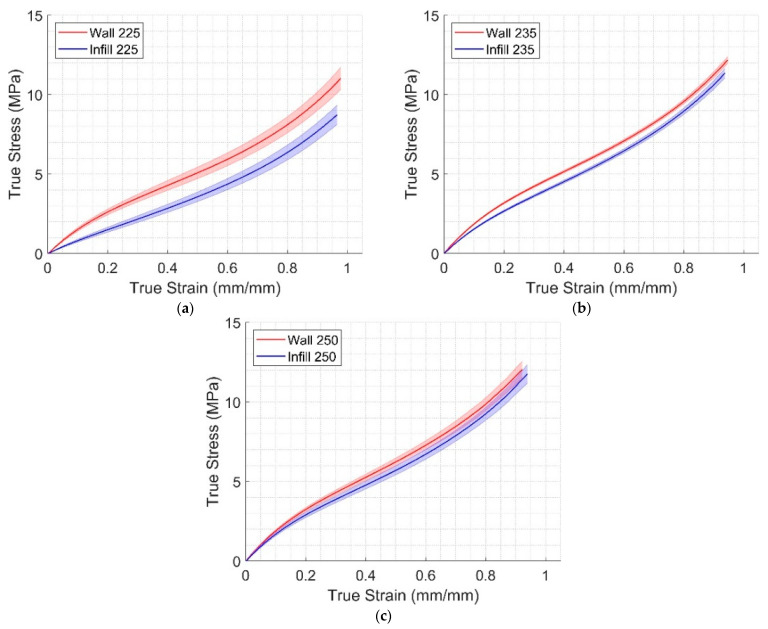
Average and standard deviation experimental results of wall-only and infill-only specimens at different extrusion temperatures, at (**a**) 225 °C; (**b**) 235 °C; and (**c**) 250 °C. The thick lines represent the average values, while the shaded regions represent the standard deviations.

The engineering stress and strain were calculated using each specimen’s dimensions. These data were then converted into their equivalent true stress and true strain. The average and standard deviation of true stress were then calculated for each group. Figure 4 shows the resulting true stress–strain curves. The average stresses of all specimen groups were compared at 0.9 true strain, listed in Table 5. The elastic modulus for all groups was also calculated using the corresponding engineering stress–strain curves based on their linear portions, which varied from 0.04 to 0.06 engineering strain.

**Figure 5:**
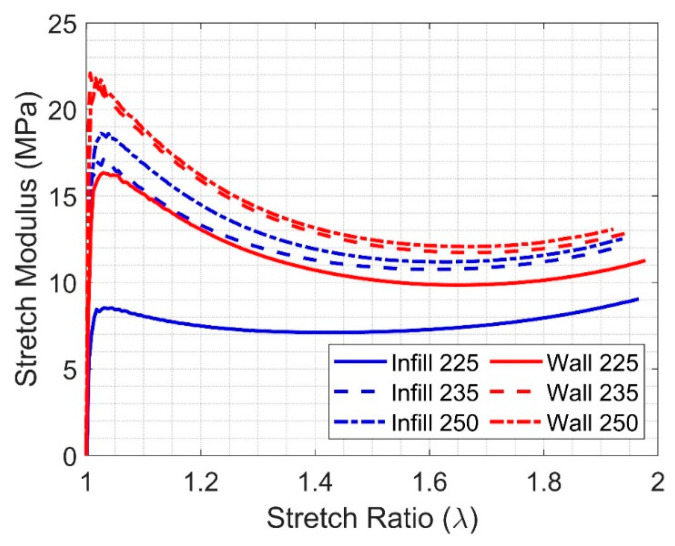
Stretch modulus of all specimen groups.

Due to the nonlinear behavior of TPUs and the narrow linear portion of the stress–strain curves, it may be appropriate to use the stretch modulus to describe the nonlinear elastic response of an isotropic hyperelastic material under uniaxial loading [37,38]. The stretch modulus can be defined as

(1) $$S=\frac{\sigma }{ln\left(\lambda \right)}=\frac{\sigma }{ln\left(1+\epsilon \right)}$$

where λ, ϵ, and σ are the principal stretch ratio, the principal true strain, and the corresponding Cauchy principal stress in the direction of loading, respectively. The stretch modulus of each group was calculated based on the average stresses and strains of each group. The results are shown in Figure 5.

### 3.2. Constitutive Model

Earlier researchers have presented possible constitutive models for TPUs. For example, it was found that second- or third-order Ogden constitutive models of Ninjaflex were accurate for strains ranging up to 550–1200% [8,39]. However, neither work has considered the effect of the material deposition method nor the extrusion temperature.

Using the material calibration software, MCalibration^®^, Version 7 (Dover, MA, USA), various constitutive models were used to find out which best fitted all specimen groups in the test matrix. In this process, each of the six groups of experimental true stress-true strain data were fitted into the constitutive model. The coefficients of each group were determined using a global optimal search to best fit the ten experimental data curves. Various orders of the constitutive model were evaluated. The coefficients of this model were determined with the goal of minimizing the normalized mean absolute difference (NMAD) fitness value.

(2) $$NMAD=\frac{\sum_{i=1}^{n}\left(\frac{\frac{\sum_{j=1}^{m_{i}}\left|\sigma_{exp_{j,i}}-\sigma_{m_{j,i}}\right|}{m_{i}}}{\max\left(\frac{\sum_{j=1}^{m_{i}}\left|\sigma_{exp_{j,i}}\right|}{m_{i}},\frac{\sum_{j=1}^{m}\left|\sigma_{m_{j,i}}\right|}{m}\right)}\right)}{n}$$

where $\sigma_{exp_{j,i}}$ and $\sigma_{m_{j,i}}$ are the vector of experimental and corresponding predicted model true stress data for trial *i*, respectively; $m_{i}$ is the number of data points on the stress–strain curve of specimen *i*; and *n* is the number of specimens.

**Figure 6:**
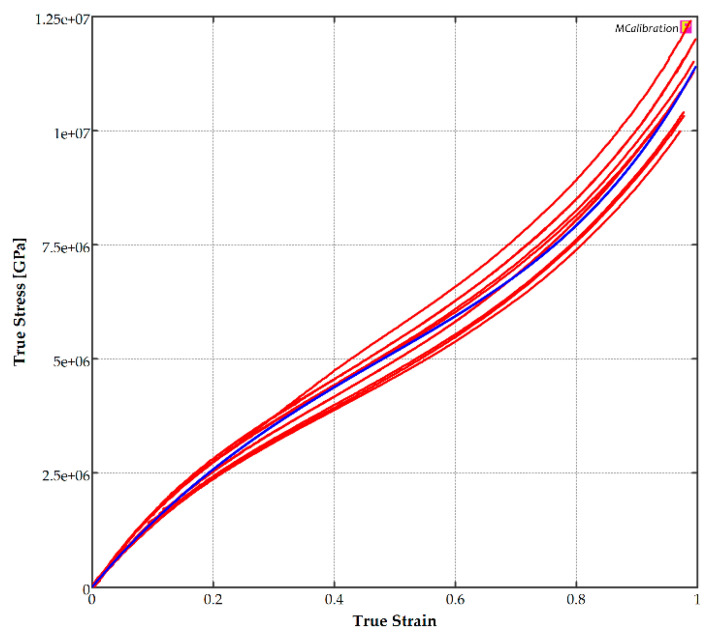
Third-order Mooney–Rivlin material curve fitting of wall-only specimens printed at 225 °C (blue), MCalibration^®^. Experimental curves of different specimens are shown in red.

It was found out that the third-order Mooney–Rivlin constitutive model [40] best represents the effect of varying FDM process parameters on TPU parts. The governing equations of this model and its application to uniaxial loading are summarized in Appendix A. The coefficients of this constitutive model, $C_{10}$, $C_{01}$, and $C_{11}$, are listed in Table 6. A representative result for the wall-only group, printed at 225 °C, is shown in Figure 6.

### 3.3. Finite Element Validation

**Figure 7:**
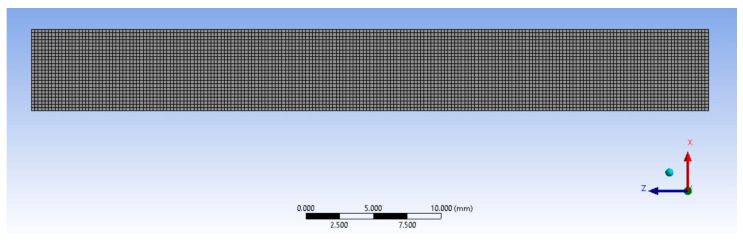
FEA mesh of the tensile specimens.

To assess the validity of the constitutive models of the previous section, FEA models were developed for each group. Based on the nominal dimensions of the specimens, Table 2 and Table 3, a planar model of the tensile specimens was created in ANSYS^®^ Mechanical (Canonsburg, PA, USA). A mesh stability study was conducted, in which element length was systematically decreased from 0.5 mm to 0.1 mm until the difference in simulation results varied by less than 1% between two adjacent element sizes. The results led to the conclusion that a mesh with an average element length of 0.25 mm (Figure 7) is sufficient. This mesh had 4800 elements. The left edge of the model was completely fixed while a ramped tensile load was applied to the right edge as a ramp. The maximum loads corresponded to the average maximum load experienced by each group, Table 7.

**Figure 8:**
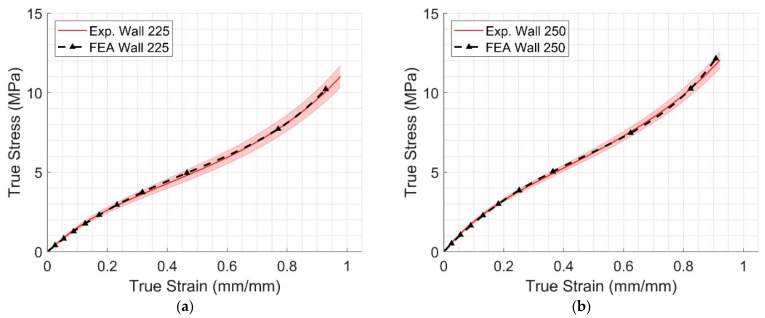
Examples of model validation results. (**a**) Wall-only specimens, extrusion temperature: 225 °C; (**b**) wall-only specimens, extrusion temperature: 250 °C.

To compare the results of the FEA models to the average of the corresponding experimental results, an error function was defined and applied to each group.

(3) $$E=\frac{\sum_{j=1}^{m}\frac{\left|\sigma_{exp_{j}}-\sigma_{FEA_{j}}\right|}{\sigma_{exp_{j}}}}{m}$$

where $\sigma_{exp_{j}}$ and $\sigma_{FEA_{j,i}}$ are the vectors of experimental and corresponding predicted model true stress data for trial *i*, respectively, and *m* is the number of points on the stress–strain curve. The error measures for each group can be found in Table 8. Typical results of the FEA model compared with the corresponding experimental data are shown in Figure 8a,b, respectively.

### 3.4. Application to Component with Multiple Regions

It is important to incorporate the proposed constitutive models within finite element simulations. While several researchers have investigated the factors that affect the modeling of 3D printed components using FEA [6,41], these studies did not consider the hyperelasticity of the 3D printed material, large deflections, or the effect the variations in the material properties of different regions.

**Figure 9:**
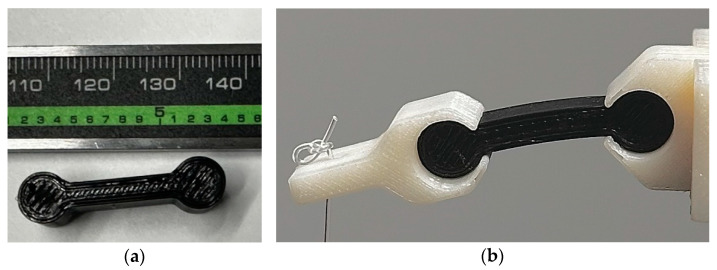
Bending experiment: (**a**) dog bone component; (**b**) sample component under deformation.

To test the proposed material models, an experiment was conducted using three identical dog bone-shaped components with three wall lines and infill material, Figure 9a. These specimens were printed at a 225 °C extrusion temperature using the same printer that was used to create the uniaxial specimens of Section 2. As expected, nominal dimensions were somehow different from actual dimensions, Table 9. These differences are small, except in the case of wall and infill thicknesses.

The specimens were loaded such that they experienced large deformations. Figure 9b shows that quasi-static loading was applied using weights that were applied to an end fixture whose mass is 3.41 g using a fishing line. The weights were incremented in 20 g steps up to 100 g.

**Figure 10:**
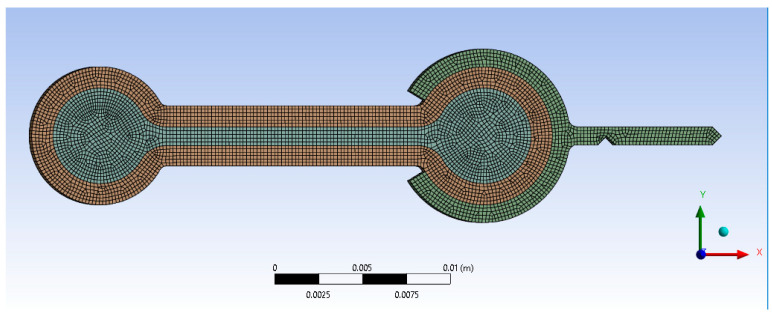
FEA mesh of the bending experiments. The model was split into three parts, with the infill region of the specimen (gray), wall region (brown), and end fixture (green).

ANSYS^®^ was used to simulate the experiment [42]. The problem was modeled using plane stress assumptions. Using the actual dimensions of Table 9, the component was split into the infill and wall regions. In addition to the external load described above, the weight of the end fixture was applied to its center of mass. A mesh stability study was conducted, and it was found that an average element size of 0.2 mm was stable, Figure 10, resulting in 4736 elements.

**Figure 11:**
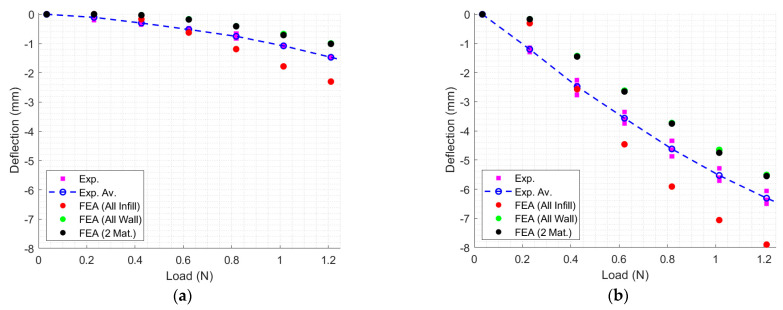
Comparison of experimental results with three FEA models, All-Infill, All-Wall, and Two Materials; (**a**) horizontal vertical displacement; (**b**) vertical displacement.

These mesh and boundary conditions were used to develop three models, namely a (i) specimen modeled using infill material for both regions (All-Infill), (ii) specimen modeled using wall material for both regions (All-Wall), and (iii) specimen modeled using infill material in the inner portion and wall of the outer portion (Two Materials). These three models used the corresponding coefficients of wall-only and infill-only constitutive models at 225 °C, Table 6. The horizontal (retraction) and vertical displacements of the neutral axis at the end of the rectangular portion of these three models were compared to the corresponding experimental data in Figure 11a,b, respectively.

## 4. Discussion

A qualitative assessment of the results shows the reasons behind the design parameters of this study, which were the extrusion temperature and material deposition method. In Figure 4a, the wall-only and infill-only curves were distinct, but they got closer in Figure 4b and were almost blended in Figure 4c. Also, at 0.9 true strain, true stress varied 7.8 MPa for infill-only and 225 °C to 11.5 MPa for wall-only and 250 °C; this difference corresponds to an almost 33% reduction in strength. These variations are typically not reported in the literature, which may lead engineers to design components based on bad assumptions. The difference in material behavior can be further inferred from Figure 5, where the stretch modulus of the infill-only at 225 °C is significantly different than the other five groups.

It is apparent from the results of Table 4 that both infill-only and wall-only specimen have voids. Infill-only samples have a consistently higher void ratio than wall-only samples, which may be a function of the way the material is deposited in each case, as wall-only specimens have filaments deposited along the length of the specimens, while infill-only specimens have filaments at ±45°. Therefore, it is expected that wall-only specimens have stronger tensile properties similar to fibers in composite materials [43]. The results also indicate the void ratios decreased by increasing the extrusion temperature, indicating that the rise in temperature fused the material better and therefore reduced the voids inside printed parts. Interestingly, the void ratios were close for wall-only specimens at 235 °C and 250 °C extrusion temperatures, which may indicate that the effect of extrusion temperature on the void ratio diminishes as it approaches the maximum recommended temperature.

The void ratio observations are consistent with the results of Table 5, as the values of the elastic moduli and the stresses at 0.9 strain were proportional to the extrusion temperatures. Similarly, infill-only specimens had higher values of these two variables when compared to wall-only specimens. Qualitatively, Figure 4 shows that the stress–strain curves of wall-only and infill-only are close to each other at 250 °C. The same behavior can be observed in the stretch modulus, Figure 5, where infill-only specimens are consistently below the wall-only and the stretch modulus of the infill-only specimens at 225 °C is significantly below other groups. These observations may suggest that using the highest recommended extrusion temperature may lead to fewer voids and better layer adhesion, as noted in [29]. The effect of the void ratio decrease at higher extrusion temperatures on strength is most apparent in the case of the infill-only. In this case, higher temperatures led to better fusion of the layers, as filaments are deposited at ±45°. This effect was less pronounced for wall-only specimens, which are deposited in the same direction.

The results of Table 6 indicate that the third-order Mooney–Rivlin constitutive model can accurately describe the behavior of FDM TPU components, confirming the observations in [44] that this model was suitable for polymers undergoing strains up to 200%. Table 6 does not exhibit any abrupt changes in the values of the three coefficients of the material model for either material deposition or extrusion temperatures. Therefore, the coefficients indicate that it is possible to interpolate between these values and obtain a material mode based on the extrusion temperature and deposition method for a TPU component. FEA validation of these experiments was conducted successfully.

**Figure 12:**
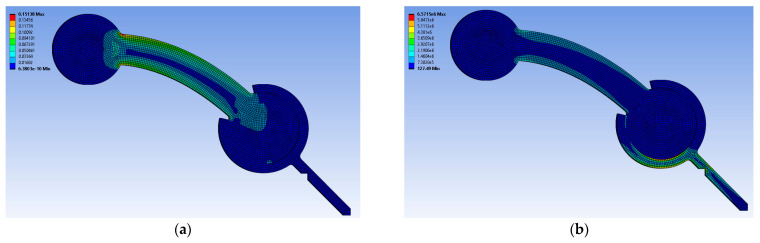
Equivalent elastic strain (**a**) and stress (**b**) of an FDM TPU component.

Finally, the constitutive materials developed in this work were applied to FDM TPU components undergoing large deformation. The FEA comparison with experiments (Figure 11) show that the Two Materials and All-Wall models are close to the experimental data’s average, except for low loads, which may indicate that the contribution of the infill portion at the specimen’s core to the overall deformation is limited. As expected, the All-Infill model overestimated the deformation. These results can be confirmed by observing Figure 12, where the highest strain and stress were observed near the left end of the rectangular portion, with strain reaching 15%, which can be compared with Figure 4a.

This work has some limitations, as the only material tested was NinjaFlex^®^. While this material is widely available, each TPU will have different material properties. This study can be expanded by considering all FDM process parameters. It is also of interest to assess the validity of this model at higher strain rates. While the results of Section 3.3 were satisfactory, it may be necessary to conduct extensive compressive testing of the material to assess how the compressive stress–strain curves differ from their tensile counterparts. The effect of using cellular or gyroid specimens should also be considered.

## 5. Conclusions

This study aims to assess the effect of FDM 3D printing process parameters, extrusion temperatures, and the filament deposition method (wall and infill) on the constitutive model of the TPU components. NinjaFlex^®^ TPU (Manheim, PA, USA) was studied. It was found that the third-order Mooney–Rivlin constitutive model can accurately describe the effect of the tested parameters. The results show that as the extrusion temperature increases, the effect of the material deposition method becomes more limited. Additionally, wall deposition leads to higher strength. Applying the constitutive models to the FEA of TPU components undergoing large deformation resulted in satisfactory accurate results when compared to the experimental results. The proposed approach can lead to better understanding of the behavior of FDM TPU parts, especially those experiencing large deformations. NinjaFlex^®^ TPU was chosen due to its commercial availability. Other TPUs should perform about as well, but we do expect some variation between different TPUs. The concepts explored in this research should be applicable to other TPUs, where different nozzle temperatures and material deposition methods may affect mechanical properties. Based on this research, the user can vary extrusion temperatures and the infill and wall ratios to obtain the desired mechanical behavior.

## Figures and Tables

**Table 1:** Three-dimensional printing parameters.

Printing Parameter	Value
Filament Diameter	1.75 mm
Infill Density	100%
Quality	Standard (0.32 mm layer height)
Bed Temperature	60 °C
Print Speed	15 mm/s
Wall Thickness	1 mm

**Table 2:** Test matrix.

Material Deposition Method	Extrusion Temperature (°C)
Infill-only	225
	235
	250
Wall-only	225
	235
	250

**Table 3:** Tensile specimen nominal dimensions.

Width, w, (mm)	Thickness, t, (mm)	Total Length, $l_{t}$, (mm)	Free Length, $l_{f}$, (mm)
6	3	115	50

**Table 4:** Measured dimensions of printed specimens (ten tensile specimens for each group).

Print Region	Extrusion Temp. (°C)	Width, w, (Std. Dev.) (mm)	Thickness, t, (Std. Dev.) (mm)	Mass, (Std. Dev.) (g)	Void Ratio
Nominal		6.0	3.0	2.5	
Infill-only	225	5.8 (0.10)	3.0 (0.06)	1.7 (0.04)	0.32
	235	5.8 (0.04)	3.0 (0.02)	2.0 (0.01)	0.20
	250	5.9 (0.02)	3.0 (0.13)	2.1 (0.02)	0.16
Wall-only	225	5.9 (0.05)	2.9 (0.04)	1.8 (0.07)	0.28
	235	5.9 (0.02)	3.1 (0.02)	2.2 (0.02)	0.12
	250	6.1 (0.17)	3.0 (0.13)	2.2 (0.02)	0.12

**Table 5:** Average mechanical properties of specimen groups.

Print Region	Extrusion Temperature (°C)	Elastic Modulus (MPa)	Stress at 0.9 True Strain (MPa)
Infill-only	225	8.14	7.69
	235	15.84	10.61
	250	17.63	10.99
Wall-only	225	15.36	9.59
	235	18.73	11.25
	250	19.48	11.62

**Table 6:** Fitted third-order Mooney–Rivlin constitutive model coefficients and fitness results.

Print Region	Extrusion Temp. (°C)	$C_{10}$ (Pa)	$C_{01}$ (Pa)	$C_{11}$ (Pa)	NMAD (%)
Infill-only	225	−2.04E5	1.64E6	6.30E4	5.97
	235	−1.40E6	4.05E6	1.95E5	1.98
	250	−1.68E6	4.56E6	2.21E5	3.18
Wall-only	225	−1.55E6	4.21E6	1.90E5	4.96
	235	−1.93E6	5.12E6	2.34E5	1.76
	250	−2.33E6	5.70E6	2.93E5	3.30

**Table 7:** Maximum tensile load for model validation.

Print Region	Extrusion Temperature (C)	Average Maximum Load (N)
Infill-only	225	58.7
	235	78.2
	250	80.8
Wall-only	225	72.7
	235	86.2
	250	88.2

**Table 8:** Error measure of the third-order Mooney–Rivlin constitutive model for each specimen group.

Print Region	Extrusion Temperature (C)	E (%)
Wall-only	225	3.02
	235	3.45
	250	5.18
Infill-only	225	4.53
	235	4.99
	250	3.46

**Table 9:** Comparison of nominal versus actual average dimensions of dog bone components (mm).

	Overall Length	Length	Height	Depth	End Diameters	Overall Thickness	Wall Thickness	Infill Thickness
Nominal	29.8	15.0	3.6	8.0	7.75	3.6	1.5	0.6
Actual	30.7	14.3	3.4	8.1	7.8	3.4	1.2	1.0

## Data Availability

Data are contained within the article.

## Appendix A

The equations for the hyperelastic TPU’s constitutive model were based on the work of Mooney and Rivlin [40]. The process started by defining the strain energy for an isotropic material undergoing large elastic strain, which can be expressed as [44,45]

(A1) $$W=\overset{N}{\underset{p,q=0}{\sum }}C_{p,q}{\left({\bar{I}}_{1}-3\right)}^{p}{\left({\bar{I}}_{2}-3\right)}^{q}+\overset{M}{\underset{m=1}{\sum }}\frac{1}{D_{m}}{\left(J-1\right)}^{2m}$$

It is sufficiently accurate at *N* = 1 for most materials. $D_{m}$ are the coefficients related to the volume changes in the material and J is the determinant of the deformation gradient. $C_{p,q}$ are the coefficients of the material model and ${\bar{I}}_{1}$ and ${\bar{I}}_{2}$ are the Cauchy strain tensor invariants.

(A2) $${\bar{I}}_{1}=J^{-\frac{2}{3}}I_{1}$$

(A3)
$${\bar{I}}_{2}=J^{-\frac{4}{3}}I_{2}$$

If the material is considered incompressible, then J = 1. Therefore, ${\bar{I}}_{1}=I_{1}$ and ${\bar{I}}_{2}=I_{2}$. The third-order constitutive model expresses the strain energy equation in terms of three coefficients, $C_{10}$, $C_{01}$, and $C_{11},$ as follows:

(A4) $$W=C_{10}\left(I_{1}-3\right)+C_{01}\left(I_{2}-3\right)+C_{11}\left(I_{1}-3\right)\left(I_{2}-3\right)$$

In the case of uniaxial loading, the principle uniaxial stress, $t_{xx}$, is defined as

(A5) $$t_{xx}=2\left(\lambda^{2}-\frac{1}{\lambda }\right)\left(\frac{\partial W}{\partial I_{1}}+\frac{1}{\lambda }\frac{\partial W}{\partial I_{2}}\right)$$

where λ is the stretch in the principal direction, and the derivatives of the strain energy with respect to the invariants are

(A6) $$\frac{\partial W}{\partial I_{1}}=C_{10}+C_{11}\left(2\lambda +\frac{1}{\lambda^{2}}-3\right)$$

(A7)
$$\frac{\partial W}{\partial I_{2}}=C_{01}+C_{11}\left(\lambda^{2}+\frac{2}{\lambda }-3\right)$$

Applying these derivatives to Equation (A5),

(A8) $$t_{xx}=2\left(\lambda^{2}-\frac{1}{\lambda }\right)\left(\left(C_{10}+C_{11}\left(2\lambda +\frac{1}{\lambda^{2}}-3\right)\right)+\frac{1}{\lambda }\left(C_{01}+C_{11}\left(\lambda^{2}+\frac{2}{\lambda }-3\right)\right)\right)$$

Equation (A8) describes the true stress as a function of stretch, λ. At this stage, the problem is reduced to determining the necessary coefficients ($C_{10}$, $C_{01}$, and $C_{11}$) that provide the best fit to the experimental data.
